# Clinical efficacy of intracavernous injection of platelet lysate for erectile dysfunction

**DOI:** 10.1186/s12894-024-01633-2

**Published:** 2024-10-29

**Authors:** Yi-Kai Chang, I-Ni Chiang, Hong-Chiang Chang, Yi-Hui Chen, Shih-Chieh Jeff Chueh

**Affiliations:** 1 Department of Urology, College of Medicine, National Taiwan University Hospital, National Taiwan University, No. 7, Zhongshan S. Rd., Zhongzheng Dist., Taipei City, 100 Taiwan; 2 College of Medicine, National Taiwan University Hospital, National Taiwan University, Taipei, Taiwan

**Keywords:** Erectile dysfunction, Platelet-rich plasma, Platelet lysate, Intracavernous injection

## Abstract

**Background:**

Among the emerging treatments for erectile dysfunction (ED), platelet-rich plasma (PRP), known for its ability to enhance tissue repair and regeneration, stands out as a promising therapeutic approach. In this innovative study, we aimed to assess the efficacy of intracavernous injections of platelet lysate (PL), a derivative of PRP, in improving erectile function among ED patients.

**Methods:**

We enrolled twenty-six patients, aged between 35 and 70 years (mean age 51.6 ± 11.3 years), who had been experiencing ED for over six months and had an International Index of Erectile Function-5 (IIEF-5) score of 21 or less. Participants received autologous PL injections intracavernously every two weeks for a total of five administrations. We assessed Erection Hardness Score (EHS) and International Index of Erectile Function-5 (IIEF-5) bi-weekly for 16 weeks and conducted penile Doppler ultrasounds pre- and post-treatment to record peak systolic velocity (PSV) and resistance index (RI).

**Results:**

Before treatment, the mean EHS was 2.15 ± 0.88 and IIEF-5 was 10.92 ± 5.28. Remarkable improvements were observed post-treatment, with the EHS significantly increasing to 3.15 ± 0.83 (*p* < 0.05) and IIEF-5 to 17.23 ± 5.26 (*p* < 0.05). Penile Doppler ultrasound exhibited an increase in both PSV and RI post-treatment, with the rise in RI being statistically significant.

**Conclusions:**

Our findings indicate that intracavernous injections of PL substantially enhance erectile function, as evidenced by improvements in EHS, IIEF-5, and the RI of penile Doppler ultrasound, without hemorrhagic events or other adverse reactions apart from temporary pain at the injection site during the 16-week follow-up period. These encouraging results suggest that PL injections are a safe and effective treatment modality for patients with moderate ED, potentially providing a less invasive and more physiologically friendly alternative to current ED management strategies.

**Trial registration:**

The study received approval from the Institutional Review Board of National Taiwan University Hospital (IRB Number 202008061RIPC, date of registration 08/28/2020).

**Supplementary Information:**

The online version contains supplementary material available at 10.1186/s12894-024-01633-2.

## Introduction

Erectile dysfunction (ED), characterized by the persistent inability to attain or sustain an erection sufficient for satisfactory sexual performance, is a multifactorial condition with both physical and psychosocial contributors. Traditional management strategies encompass lifestyle adjustments, cardiovascular exercise, administration of phosphodiesterase-5 (PDE5) inhibitors with differing pharmacokinetics, vacuum erection devices, intracavernous alprostadil injections, and penile prosthesis implantation. In a breakthrough for regenerative medicine, low-intensity shock wave therapy has been introduced as an innovative treatment, primarily targeting vasculogenic ED due to its potential to enhance penile blood flow and stimulate neovascularization [1].

In recent years, a surge in both preclinical and clinical research has focused on the therapeutic potential of platelet-rich plasma (PRP) in the realm of ED. PRP, a preparation obtained from centrifuged autologous blood, is essentially a concentrated source of platelets, encompassing various growth factors and cytokines known to augment tissue regeneration and healing [2]. An advanced form of PRP is platelet lysate (PL), achieved by lysing the platelets, thereby ensuring the comprehensive release of their reservoir of growth factors. This configuration not only maximizes the availability of growth factors but also maintains an exceedingly low white blood cell count, less than 1%, minimizing the risk of immune reactions and inflammatory responses. PL has been reported to be effective in treating osteoarthritis, radicular pain, and promoting wound healing due to its high concentration of growth factors and low levels of white blood cells, which contribute to accelerated tissue regeneration, enhanced wound healing, and reduced inflammatory response [3].

Recent studies have illuminated the promise of PRP and its derivatives in ED management. For instance, a systematic review by Mohammad et al. demonstrated that PRP therapy could significantly enhance erectile function scores in men, paralleling the effects of other common ED treatments without accompanying systemic side effects [4]. Similarly, a review and study in 2022 consolidated evidence from multiple trials, underscoring the potential benefits of PRP injections in improving erectile function, particularly among patients with vasculogenic ED [5, 6].

Furthermore, a 2023 study by Shaher et al. investigated the safety and efficacy of PRP injections in men with ED and concluded that the treatment was associated with significant improvements in erectile function and penile hemodynamics, without any serious adverse events [7]. In another compelling study by Tai et al. in 2023, the findings suggested a potential role for PRP therapy not only in managing ED in rat model but also in potentially reversing underlying pathophysiological mechanisms, emphasizing rat the regenerative capacity of PRP [8]. Additionally, a few research papers provided evidence supporting the concept that combining PRP therapy with other regenerative strategies, such as stem cell therapy or shock wave treatment, might offer synergistic benefits, paving the way for comprehensive regenerative therapeutic protocols for ED [9, 10].

In the context of these scientific advances and the potential of regenerative medicine, our study aims to explore the efficacy of intracavernous injections of PL in treating ED. We assessed this by comparing pre- and post-treatment Erection Hardness Score (EHS), the International Index of Erectile Function-5 (IIEF-5), and penile Doppler ultrasound findings, seeking not only to corroborate the findings of previous research but also to contribute new insights into the evolving landscape of ED therapy.

## Materials and methods

### Patient selection and ethical approval

**Fig. 1:**
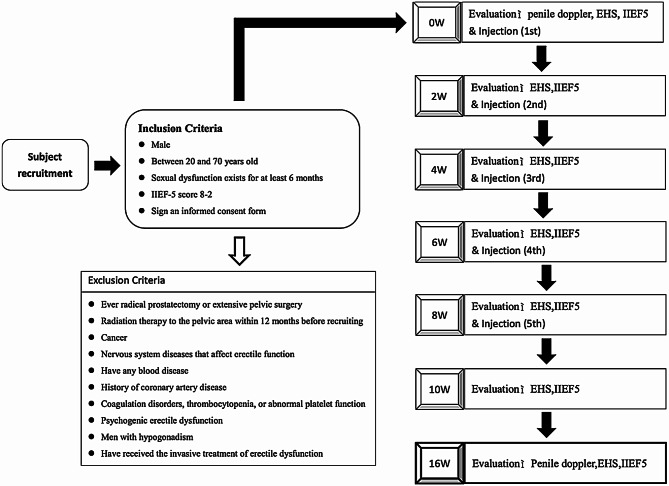
Flow diagram regarding the experimental procedure and criteria of inclusion and exclusion

The study received approval from the Institutional Review Board of National Taiwan University Hospital (IRB Number 202008061RIPC). Inform consent were given and signed by all the patients. Male participants experiencing erectile dysfunction for more than 6 months, with an International Index of Erectile Function-5 (IIEF-5) score of ≤ 21 and aged between 30 and 70 years, were enrolled for the platelet lysates (PL) study. Exclusion criteria included coagulopathy, prior penile surgery or trauma, pelvic radiation therapy within the last 12 months, previous pelvic surgery, and psychogenic erectile dysfunction (Fig. 1).

### Platelet lysates (PL) preparation

Twenty mL of peripheral blood was collected from each patient and infused into Aeon CPKit tubes (Aeon Biotherapeutics Corp., Taipei, Taiwan) in each session. Tubes filled with 10 mL of blood were then centrifuged at 1650 g for 8 min and gently inverted repeatedly. Tube filled with 10 mL of blood was then centrifuged at 1650 g for 8 min and gently inverted repeatedly. The tubes were then centrifuged multiple times at 1650 g for 8 min until platelet-rich fibrin formed. Centrifugation was performed 1 to 2 times until the platelet-rich fibrin was compressed into a thin layer on top of the separation gel, and PL was released from the fibrin. Eight to 10 mL of PL was collected from 2 tubes and prepared for injection.

### Platelet lysates (PL) injection

**Fig. 2:**
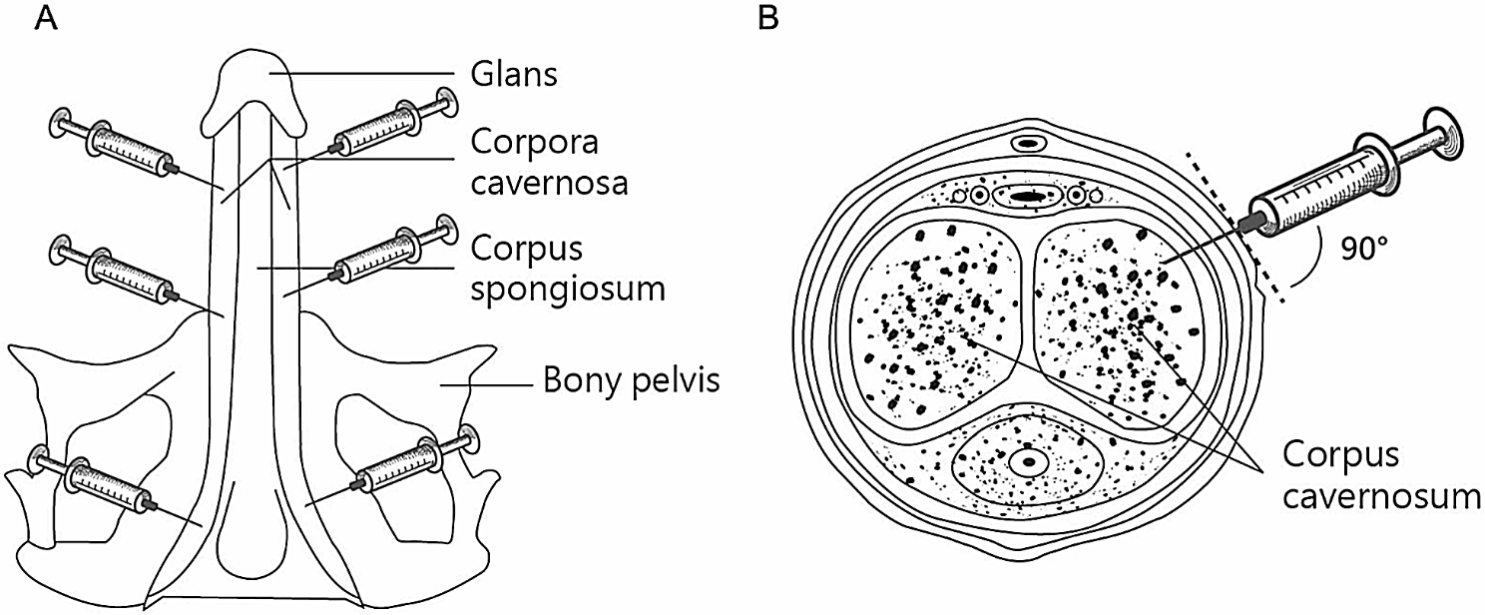
Schematic representative of platelet lysate (PL) injections. Six Injection sites viewed from underneath of penis indicate by syringe icons (**A**). Injection is shown in cross-section view of penis (**B**)

Autologous PL was administered every two weeks for a total of 5 sessions. All injections were performed by the same clinician. Eight to 10 mL of PL was injected using a 30-gauge needle at equal proportions into the bilateral distal, middle, and proximal corpus cavernosum, totaling 6 injection sites (Fig. 2).

### Penile doppler ultrasound

**Fig. 3:**
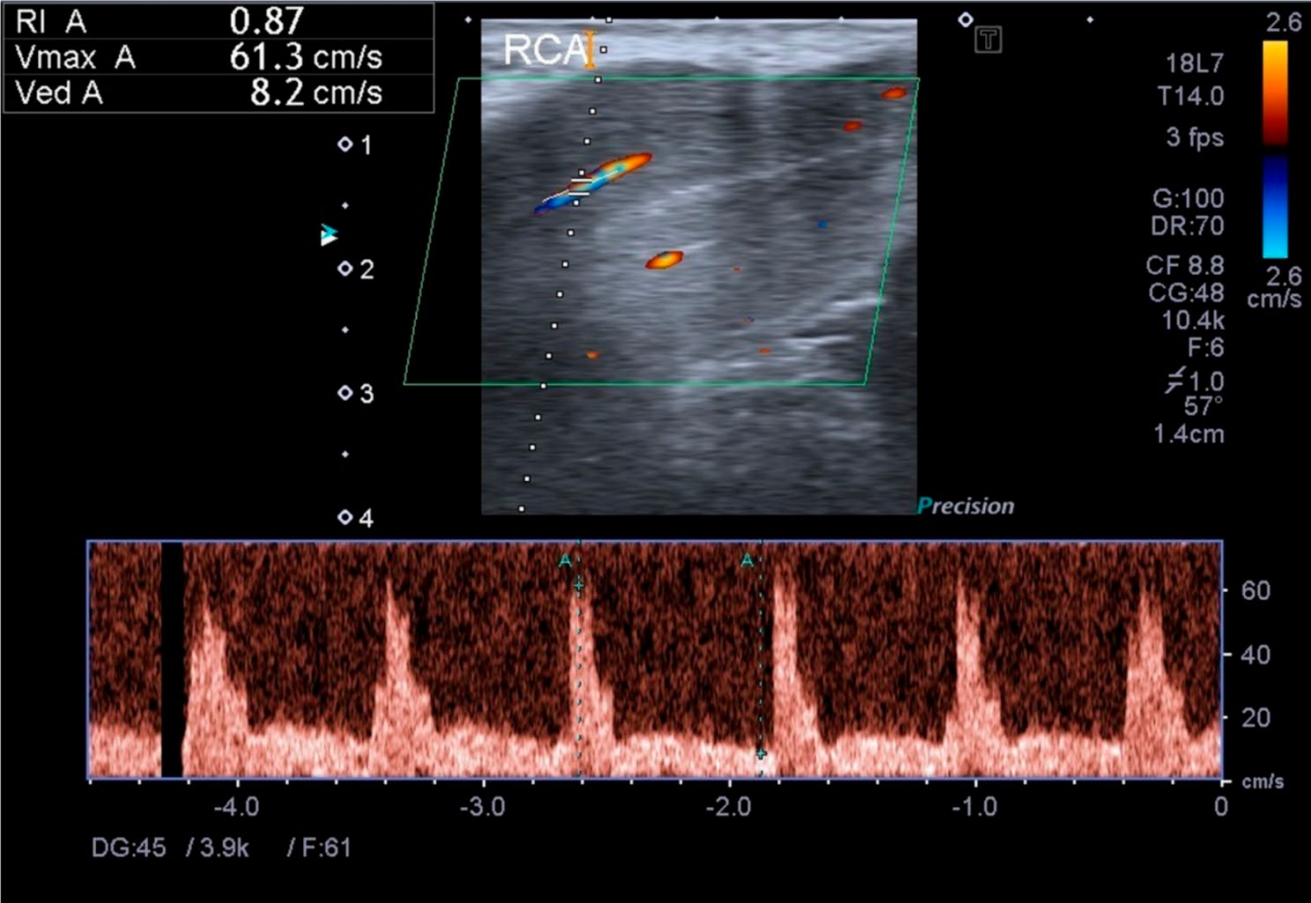
Doppler ultrasound Images of vascular flow in penis

Penile Doppler ultrasound with intracavernous alprostadil injection was conducted pre-treatment and 16-week post-treatment (Fig. 3).

### Data analysis: questionnaire and penile doppler ultrasound

The pre-treatment evaluation included the Erection Hardness Score (EHS), IIEF-5, and penile Doppler ultrasound with peak systolic velocity (PSV) and resistance index (RI). EHS and IIEF-5 were assessed every two weeks for 16 weeks. Two months after the 5th PL treatment, patients were also evaluated with post-treatment penile Doppler ultrasound. All data differences were analyzed using one-way ANOVA, and differences between groups were assessed using paired t-tests. A P value of < 0.05 was considered statistically significant.

## Results

Between 2021 and 2022, a total of 30 patients with erectile dysfunction participated in the PL treatment study. Four patients were excluded due to a history of previous penile surgery, penile trauma, or current use of antipsychotics.

**Fig. 4:**
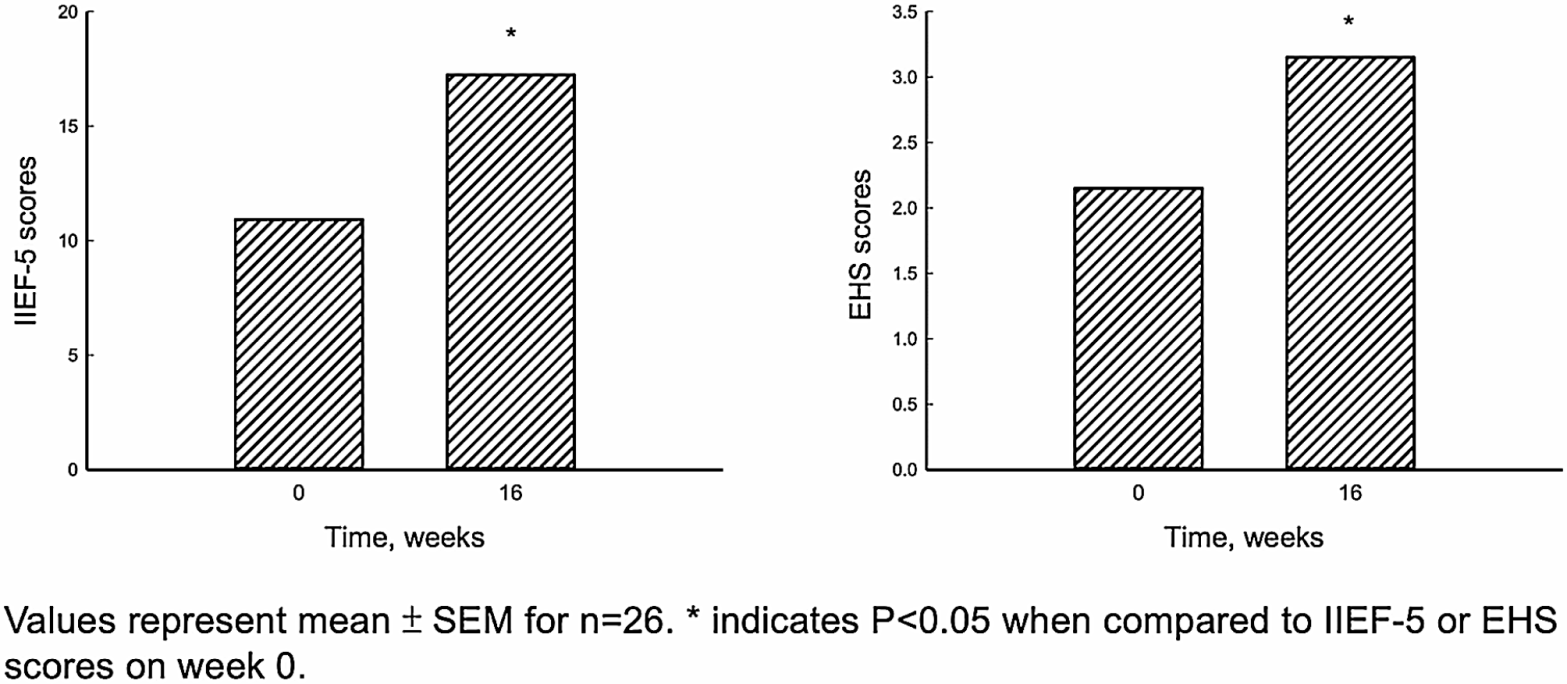
International Index of Erectile Function (IIEF-5) (**A**) and Erection Hardness Score (EHS) (**B**) scores on week 0 and 16 after PL injections

Among the 26 patients who received five intracavernous injections of PL, the mean age was 51.6 ± 11.3 years (ranging from 35 to 70 years). Pre-treatment mean Erection Hardness Score (EHS) and International Index of Erectile Function-5 (IIEF-5) were 2.15 ± 0.88 and 10.92 ± 5.28, respectively. Following treatment, EHS increased significantly to 3.15 ± 0.83 (*p* < 0.05), and IIEF-5 increased to 17.23 ± 5.26 (*p* < 0.05) (Table 1; Fig. 4). Subjectively, 80.7% of patients reported an improvement in sexual performance, describing enhancements such as easier erection, increased hardness, prolonged erection, more morning erections, easier maintenance of erection, decreased plaque, and increased girth. Among patients with improvement, pretreatment IIEF-5 ranged from 4 to 21, while those without improvement had a pretreatment IIEF-5 ranging from 5 to 10. Considering patient characteristics, including hyperlipidemia, hypertension, diabetes mellitus, and testosterone levels, only hyperlipidemia showed a negative impact on PL treatment.

**Table 1:** Patients’ characteristics

	Total	Positive	Negative	*P*-Value
N	26	21	5	
Age	51.6 ± 2.3	51.1 ± 2.5	53.4 ± 5.5	0.7
Duration of ED (Year)	4.7 ± 1.2	5.09 ± 0.5	1.8 ± 0.4	0.24
BMI	24.8 ± 0.5	24.5 ± 0.5	26.1 ± 1.4	0.26
Testosterone	4.7 ± 1.2	5.4 ± 1.5	3.4 ± 1.0	0.15
Benign prostatic hyperplasia	12	9	3	0.51
Hypertension	7	6	1	0.71
Smoking	6	5	2	0.33
Hyperlipidemia	9	5	4	0.016 *
CVA	1	1	0	0.635
CAD	3	3	0	0.38
DM	2	2	0	0.492

(All data represent mean ± SEM)

**Fig. 5:**
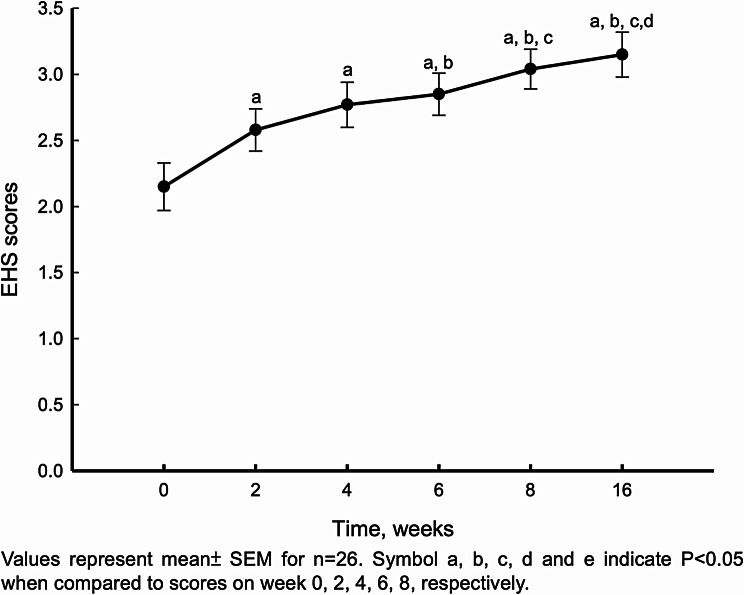
EHS scores on week 0, 2, 4, 6, 8 and 16 after PL injections

**Fig. 6:**
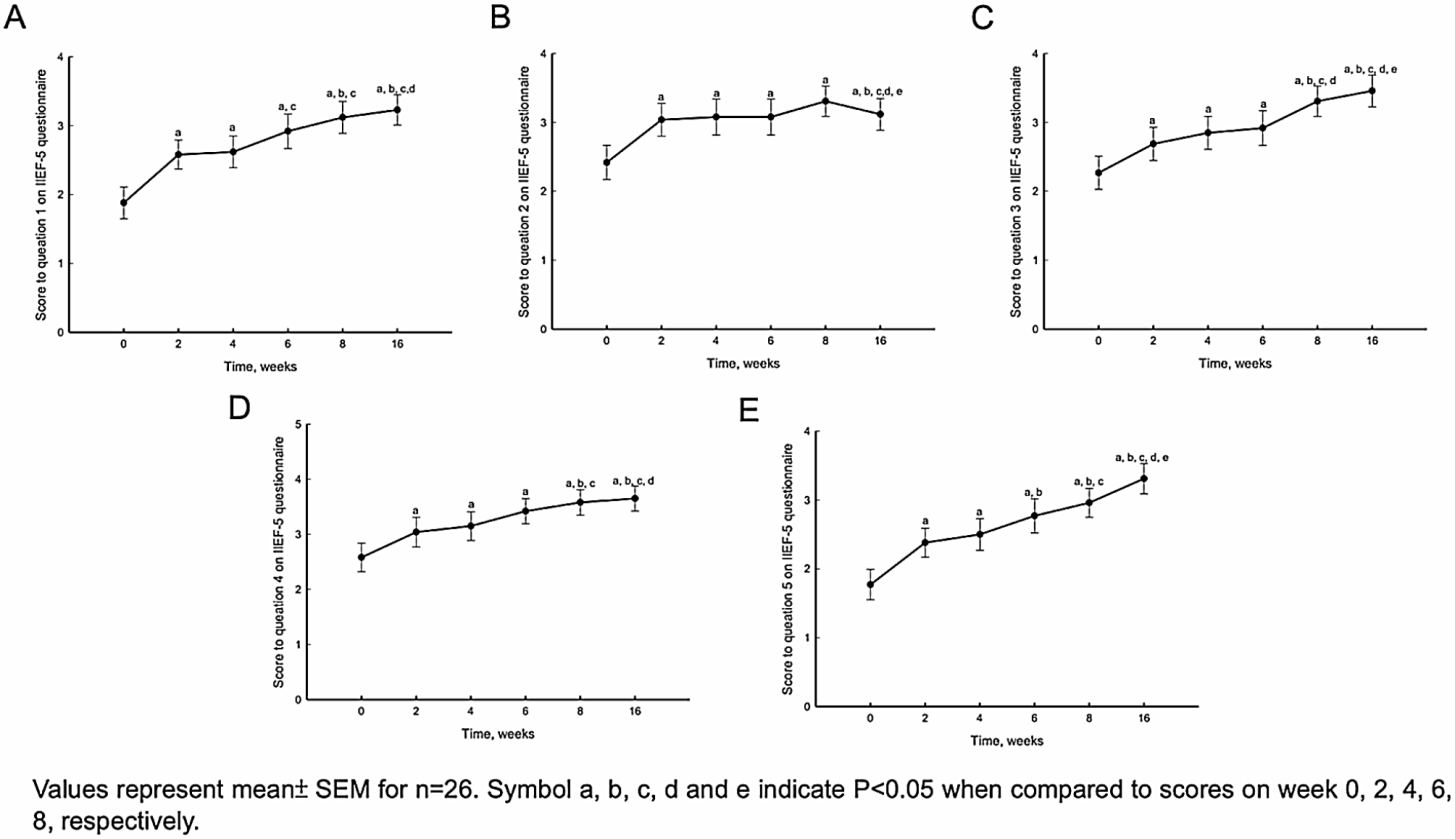
Scores to question 1 to 5 in IIEF-5 questionnaire on week 0, 2, 4, 6, 8 and 16 after PL injections (**A**, **B**, **C**, **D**, and **E**)

**Fig. 7:**
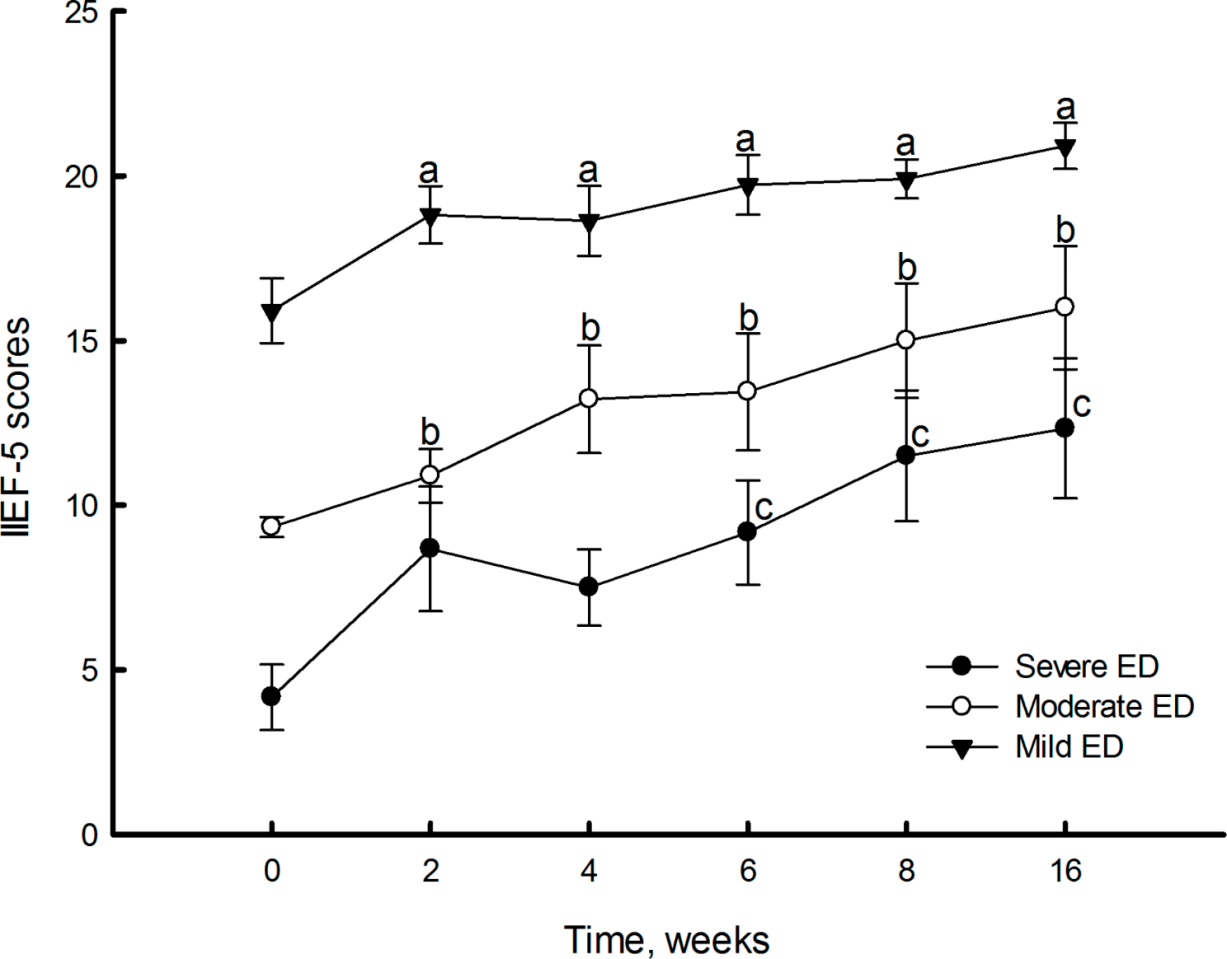
IIEF-5 questionnaire scores at weeks 0, 2, 4, 6, 8, and 16 following PL injections in patients with mild, moderate, or severe ED. Values represent the mean ± SEM for *n* = 11, 9, and 6 in patients with mild, moderate, or severe ED, respectively. Symbols a, b, and c indicate *P* < 0.05 when compared to week 0 scores in patients with mild, moderate, or severe ED, respectively

Both EHS and IIEF-5 showed significant improvement two weeks after the first PL injection (Figs. 4 and 5). Furthermore, EHS and IIEF-5 continued to gradually improve with repeated injections (Fig. 6; Table 2). We subdivided the initial IIEF score to mild, moderate, and severe ED and found IIEF-score increased in all groups. The patients with mild to moderate ED improved with one injection of PL. The patients with severe ED improved after at least three injections (Fig. 7). The details of IIEF-score were listed in Table 2.

**Table 2:**
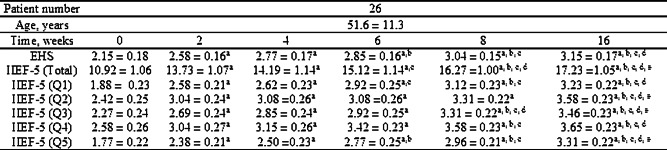
EHS score and scores to each questions in IIEF-5 questionnaire on week 0, 2, 4, 6, 8,16 after PL injections. All data represent mean ± SEM(n=26).Symbol a, b, c, d and e indicate P<0.05 when compared to scores on week 0, 2, 4, 6, 8, respectively

Post-treatment peak systolic velocity (PSV) and resistance index (RI) from penile Doppler ultrasound both increased compared to pre-treatment values, with RI showing statistically significant improvement (Table 3). No hemorrhagic events or other side effects were observed.

**Table 3:** Resistant index (RI) and peak systolic velocity (PSV) were measured and analyzed by Doppler ultrasound on week 0 and 16 after PL injections

Time, weeks	0	16	*P*-value
RI	0.81 ± 0.02	0.87 ± 0.02	0.01
PSV	39.39 ± 3.12	46.41 ± 3.62	0.07

(Each value is represented by mean ± SEM for *n* = 26. *P* < 0.05 indicates significant difference from the value of week 0.)

## Discussion

Our study demonstrated a substantial increase in International Index of Erectile Function-5 (IIEF-5) scores (by an average of 6.31 points) in patients after five sessions of PL injections. Notably, there was no deterioration in Erection Hardness Score (EHS) or IIEF-5, and no complications were reported post-injection. Patients with mild to moderate ED showed improvements after the first injection of PL, while those with severe ED experienced improvements after the third injection. Continuous improvements were observed in patients with mild, moderate, or severe ED. In consideration of patients’ characteristics, hyperlipidemia showed significant negative effects on PL therapeutic effects for ED. Patients with ED should receive at least 3 to 5 PL injections to achieve satisfactory outcomes, although hyperlipidemia may compromise these results. This offers valuable clinical insights for physicians considering PL cavernous intervention for ED patients. Doppler ultrasound analyses also indicated significant improvements in the Resistive Index (RI) following the treatment sessions. In summary, PL injections have proven effective in enhancing erectile function from both objective and subjective standpoints, thereby improving the overall quality of life for patients with erectile dysfunction.

Today, innovative treatments such as Platelet-Rich Plasma (PRP), Low-Intensity Shockwave Therapy (Li-SWT), and stem cell therapy are at the forefront of erectile dysfunction (ED) management [11, 12]. PRP is procured from blood and contains platelet concentrations that are significantly higher, about 2 to 7 times, than that found in normal plasma, making it rich in growth factors [13, 14].

The therapeutic potential of blood-derived PRP and PL in regenerative medicine is noteworthy, given the composition of platelets’ releasates. These include adhesive proteins, angiogenic factors, growth factors, chemokines, clotting factors, inhibitors, integral membrane proteins, immune mediators, and other bioactive substances. Key among these are platelet-derived growth factors (PDGF), transforming growth factor-β (TGF-β), insulin-like growth factor (IGF), fibroblast growth factor-2 (FGF-2), and vascular endothelial growth factor (VEGF) [15]. These growth factors are integral for processes like wound healing and angiogenesis, the growth of new blood vessels [16].

In the field of clinical urology, the application of PRP has transcended experimental phases, showing promise in the treatment of conditions like erectile dysfunction [17], both male and female stress urinary incontinence [18, 19], persistent BK virus-induced hemorrhagic cystitis [20], and more. PRP has demonstrated efficacy in mid-shaft hypospadias repair by reducing urethral stenosis and post-operative infection rates [21], and in offering protection against recurrent urinary tract infections by improving the expression of cytoskeletal and barrier function proteins, including CD34, Shh, CK20, M2, and M3 [22]. Remarkably, patients with interstitial cystitis showed significant symptom improvement and a change in urinary markers after receiving PRP injections [23]. Experimental models also indicate the potential of PRP in mitigating urethral stricture by reducing mucosal inflammation and spongiofibrosis [24], protecting rat testes from ischemia-reperfusion injury [25], and even providing renal protection in cases of obstructive uropathy [26].

Animal studies further substantiate the regenerative properties of PRP. Ding et al. observed that PRP facilitated cavernous nerve regeneration, improved intracavernous pressure, and increased the presence of myelinated axons and NADPH-diaphorase-positive nerve fibers in a rat model [27]. Another study demonstrated that regular intracavernous PRP injections over four weeks improved erectile function parameters in hyperlipidemia rats, outperforming the group without PRP treatment [28]. Additionally, Wu et al. reported that PRP injections post-bilateral cavernous nerve injury enhanced erectile function recovery and decreased cellular apoptosis [29]. Further, an optimized PRP preparation protocol showed superior therapeutic outcomes in tissue recovery [30], and high levels of C-X-C motif chemokine ligand 5 (CXCL5) in PRP were found to ameliorate ED by preventing penile smooth muscle atrophy [31].

Transitioning from animal models to human application, Matz et al. reported positive outcomes with PRP injections in patients with ED and Peyronie’s disease between 2012 and 2017, noting improvements in IIEF-5 scores without any significant complications other than injection-site pain [2]. Other studies, including those by Zaghloul et al. and Poulios et al., echoed these findings, showing significant improvements in IIEF scores in patients with ED who were refractory to traditional phosphodiesterase-5 (PDE5) inhibitors [32, 33]. Furthermore, Geyik et al. suggested that combining Li-SWT with PRP could enhance treatment outcomes, including a prolonged intravaginal ejaculatory latency time (IELT) [10].

The regenerative capabilities of PRP, which promote angiogenesis and tissue repair, are particularly relevant in the context of ED, often caused by inadequate blood flow or nerve damage. Huang et al. [28] conducted a comprehensive study, which underscored the potential of PRP in treating ED, particularly due to its angiogenic properties. The study suggested that the growth factors in PRP could reverse endothelial dysfunction, a common contributor to ED.

Moreover, the safety profile of PRP is an essential aspect of its applicability. A systematic review by Panunzio et al. [34] in 2023 emphasized the minimal invasiveness and low adverse event rates of PRP injections, making them a compelling treatment option for patients who might not respond to conventional therapies or for those who are seeking alternatives to pharmacological treatments.

The synergy between PRP therapy and other ED treatments also warrants discussion. Towe et al. [35] studied the combination of PRP with other regenerative therapies, including stem cells and shockwave therapy, and found that the combined treatment modalities could potentially have a synergistic effect, offering a new avenue for patients who have not responded to singular treatment modalities.

Despite the promising results of PRP therapy in preclinical studies and clinical trials, there remains a need for larger-scale, randomized, double-blinded, placebo-controlled studies to validate the efficacy and safety of PRP in the management of ED. A large-scale randomized controlled trial should be considered and conducted to evaluate the results of this study. A study by Fadadu et al. [36] emphasized the importance of standardization in platelet-rich plasma (PRP) preparation methods. They highlighted the significant variability in these methods, which can impact the concentration of platelets and levels of certain growth factors, potentially influencing the outcomes of treatments.

Finally, the cost-effectiveness of PRP treatments is an important consideration for widespread adoption. Britt et al. [37] examined the economic aspects of PRP therapy for the management of ED and concluded that although PRP therapy holds promise, the associated costs of the procedure are substantial. The authors advocate for larger-scale randomized control trials to assess the long-term efficacy of PRP in managing ED. Such trials may provide essential information to determine whether PRP should be considered a routine treatment.

## Conclusion

In conclusion, while our study and others highlight the potential benefits of PRP and PL therapies in managing ED, further investigations are needed to standardize treatment protocols, assess long-term efficacy and safety, evaluate synergy with other treatments, and analyze cost-effectiveness. As these therapies continue to evolve, they may offer new hope for patients with ED, particularly those for whom conventional treatments are ineffective, contraindicated, or unwanted.

## Electronic supplementary material

Below is the link to the electronic supplementary material.

Supplementary Material 1: Additional file 1. File format: .xls. Title of data: Table 2. EHS score and scores to each question in IIEF-5 questionnaire on week 0, 2, 4, 6, 8,16 after PL injections. Description of data: All data represent mean ± SEM(n=26). Symbol a, b, c, d and e indicate P<0.05 when compared to scores on week 0, 2, 4, 6, 8, respectively.

Supplementary Material 2

Supplementary Material 3

## Data Availability

Data is provided within the supplementary information files.

## Abbreviations

ED: Erectile Dysfunction
PRP: Platelet-Rich Plasma
PL: Platelet Lysate
EHS: Erection Hardness Score
IIEF-5: International Index of Erectile Function-5
PSV: Peak Systolic Velocity
RI: Resistance Index
PDE5: Phosphodiesterase-5
Li-SWT: Low-Intensity Shockwave Therapy
PDGF: Platelet-Derived Growth Factors
TGF-β: Transforming Growth Factor-β
IGF: Insulin-like Growth Factor
FGF-2: Fibroblast Growth Factor-2
VEGF: Vascular Endothelial Growth Factor
CXCL5: C-X-C motif chemokine ligand 5
IELT: Intravaginal Ejaculatory Latency Time
