# Supplementary figures and images for: Clinical efficacy of intracavernous injection of platelet lysate for erectile dysfunction
Source: BMC Urol. 2024 Oct 29;24:237. doi: 10.1186/s12894-024-01633-2 (PMC11520801; doi:10.1186/s12894-024-01633-2)

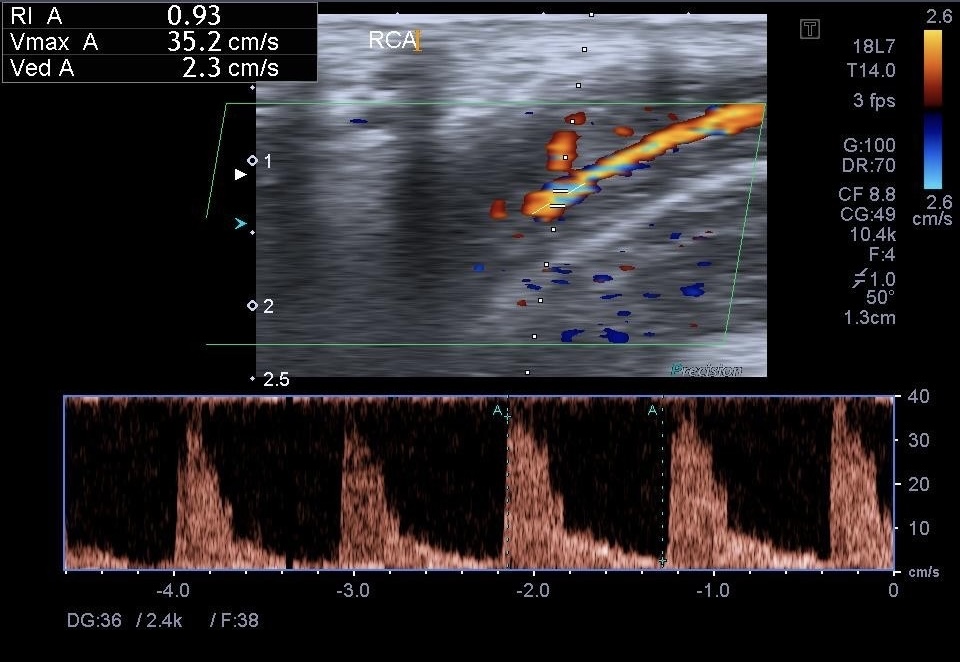

Supplement: Supplementary file 2 — Supplementary Material 2 [file 12894_2024_1633_MOESM2_ESM.jpeg]

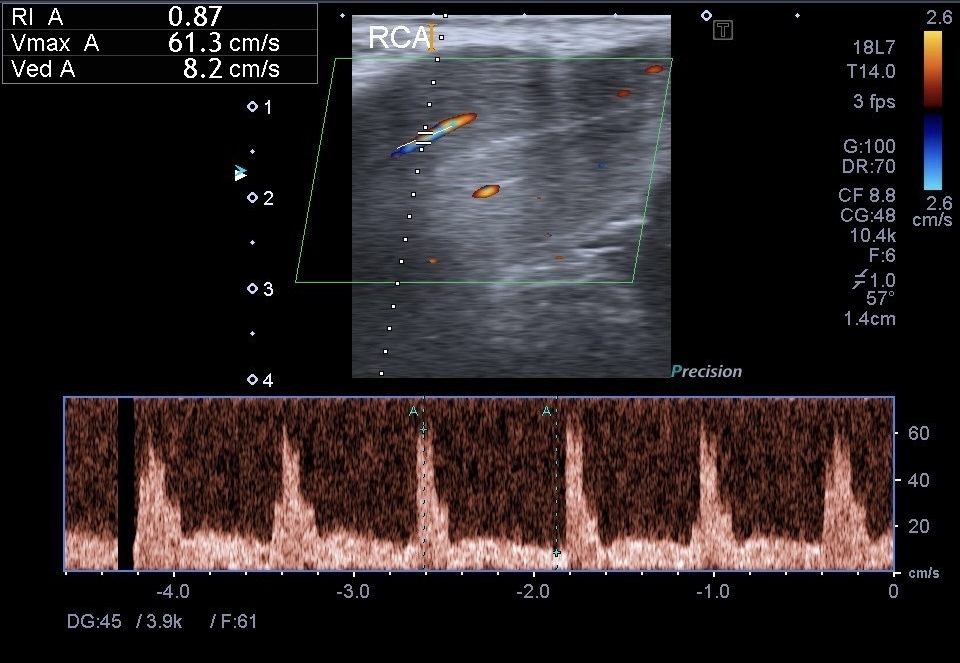

Supplement: Supplementary file 3 — Supplementary Material 3 [file 12894_2024_1633_MOESM3_ESM.jpeg]
